# Role of BAG5 in Protein Quality Control: Double-Edged Sword?

**DOI:** 10.3389/fragi.2022.844168

**Published:** 2022-03-03

**Authors:** Manish K. Gupta, Puneet Kaur Randhawa, Michal M. Masternak

**Affiliations:** Division of Metabolic and Cardiovascular Sciences, Burnett School of Biomedical Sciences, College of Medicine, University of Central Florida, Orlando, FL, United States

**Keywords:** BAG5, mitophagy, cardiovascular, Hsp70, autophagy, chaperone, aging, neuroprotection

## Abstract

Cardiovascular disorder is the major health burden and cause of death among individuals worldwide. As the cardiomyocytes lack the ability for self-renewal, it is utmost necessary to surveil the protein quality in the cells. The Bcl-2 associated anthanogene protein (BAG) family and molecular chaperones (HSP70, HSP90) actively participate in maintaining cellular protein quality control (PQC) to limit cellular dysfunction in the cells. The BAG family contains a unique BAG domain which facilitates their interaction with the ATPase domain of the heat shock protein 70 (HSP70) to assist in protein folding. Among the BAG family members (BAG1-6), BAG5 protein is unique since it has five domains in tandem, and the binding of BD5 induces certain conformational changes in the nucleotide-binding domain (NBD) of HSP70 such that it loses its affinity for binding to ADP and results in enhanced protein refolding activity of HSP70. In this review, we shall describe the role of BAG5 in modulating mitophagy, endoplasmic stress, and cellular viability. Also, we have highlighted the interaction of BAG5 with other proteins, including PINK, DJ-1, CHIP, and their role in cellular PQC. Apart from this, we have described the role of BAG5 in cellular metabolism and aging.

**GRAPHICAL ABSTRACT:**
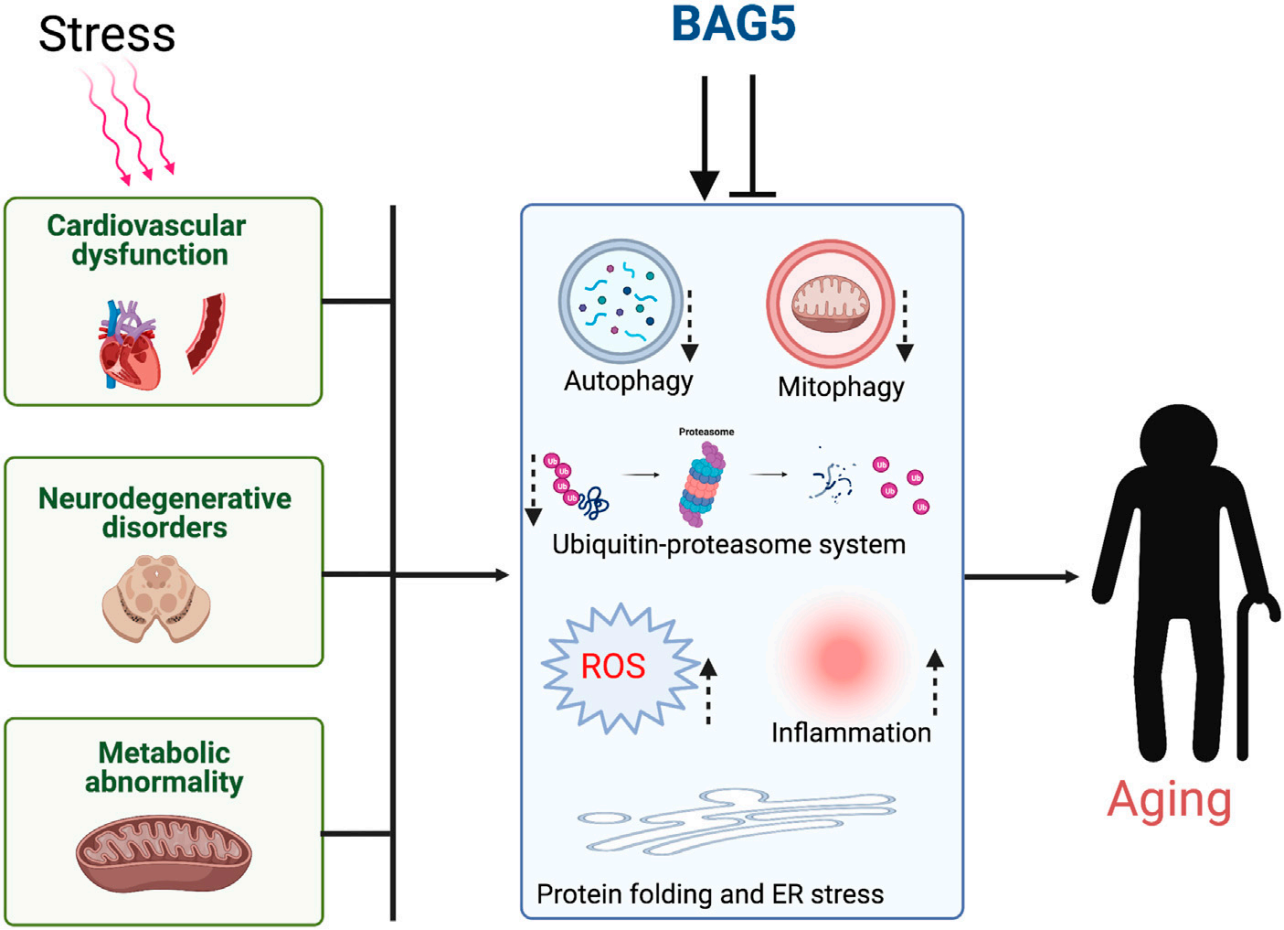
Cellular stress dysregulates the major organ function through inhibition of cellular protein quality control and induces aging.

## Introduction

Cardiovascular diseases (CVDs), including ischemic heart disease, heart failure, or other vascular conditions, constitute the leading cause of mortality among individuals worldwide (Pinto et al., 2021; Virani et al., 2021). According to the World Health Organization estimation, in the year 2019, 18.6 million people died due to CVDs globally (Randhawa and Gupta, 2020; Virani et al., 2021). Although we have made considerable progress in the cardiovascular field, there is a dire need to develop treatment strategies to treat CVDs in a fundamental way rather than in a symptomatic way. Cellular function and viability depend on the quality and quantity of cellular protein, and cellular protein quality control (PQC) plays an active role in maintaining cellular proteostasis (Henning and Brundel, 2017; Hohfeld et al., 2021). Cellular chaperones and co-chaperones actively maintain the cellular PQC by helping nascent protein fold properly and translocating the folded protein to correct destinations (Santra et al., 2019; Hartl et al., 2011). Additionally, chaperones and co-chaperones help in the removal and recycling of misfolded protein by cellular protein degradation systems. Most of the cellular protein gets degraded through the ubiquitin-proteasome system (UPS) (Sun-Wang et al., 2020; Li et al., 2021). The cell also uses a vesicle-mediated protein degradation system called autophagy to degrade cellular proteins and subcellular organelles (Delbridge et al., 2017). In addition, to these processes, misfolded proteins in the endoplasmic reticulum (ER) also get degraded through ER-associated protein degradation (ERAD) system. However, genetic mutation, hemodynamic stress, cellular toxicity, and cellular infection change cellular proteostasis and dysregulate a quintessential environment required for normal cellular metabolism. Impairment of cellular PQC and accumulation of protein aggregates with faulty subcellular organelles leads to the generation of oxidative stress, inflammation, and cell death (Li et al., 2021). These modifications are known to be associated with the impairment of several organ functions and the development of several life-threatening diseases including, CVD, neurological disorders, and premature aging (Gong et al., 2016; Kaur et al., 2020). This review will discuss the evolving role of co-chaperone Bcl-2 associated anthanogene protein (BAG5) in UPS, autophagy, mitophagy, oxidative stress, metabolism, and its association with CVDs, Alzheimer’s disease, and Parkinson’s disease.

## Role of Protein Quality Control in Heart Disease

The adult mammalian cardiomyocytes are post-mitotic cells that lack self-renewal ability (Lázár et al., 2017). Therefore, it is critical to maintain protein homeostasis and monitor PQC to limit cellular dysfunction in the cardiomyocytes (Wang and Robbins, 2006; Maejima, 2020). The maintenance of protein homeostasis includes a range of events, including gene transcription, translation, post-translational modification, complex formation, and protein degradation (Webster et al., 2020). Cellular PQC is maintained *via* two major mechanisms, the UPS and or autophagy, to eliminate misfolded proteins and damaged organelles (Maejima, 2020). Also, the ERAD system tags the unfolded proteins of ER with ubiquitin and facilitates the degradation of proteins through the UPS pathway. Studies have indicated that the accumulation of misfolded proteins may impart mechanical stress and also induce oxidative stress in the cardiomyocytes such that it may lead to the development of heart diseases, including myocardial infarction and even heart failure (Wang and Robbins, 2006; Maejima, 2020). Therefore, the maintenance of protein integrity and quality control in the cell is crucial for the normal functioning of the myocardium.

## Molecular Chaperones in Maintaining Cellular Proteostasis

**FIGURE 1:**
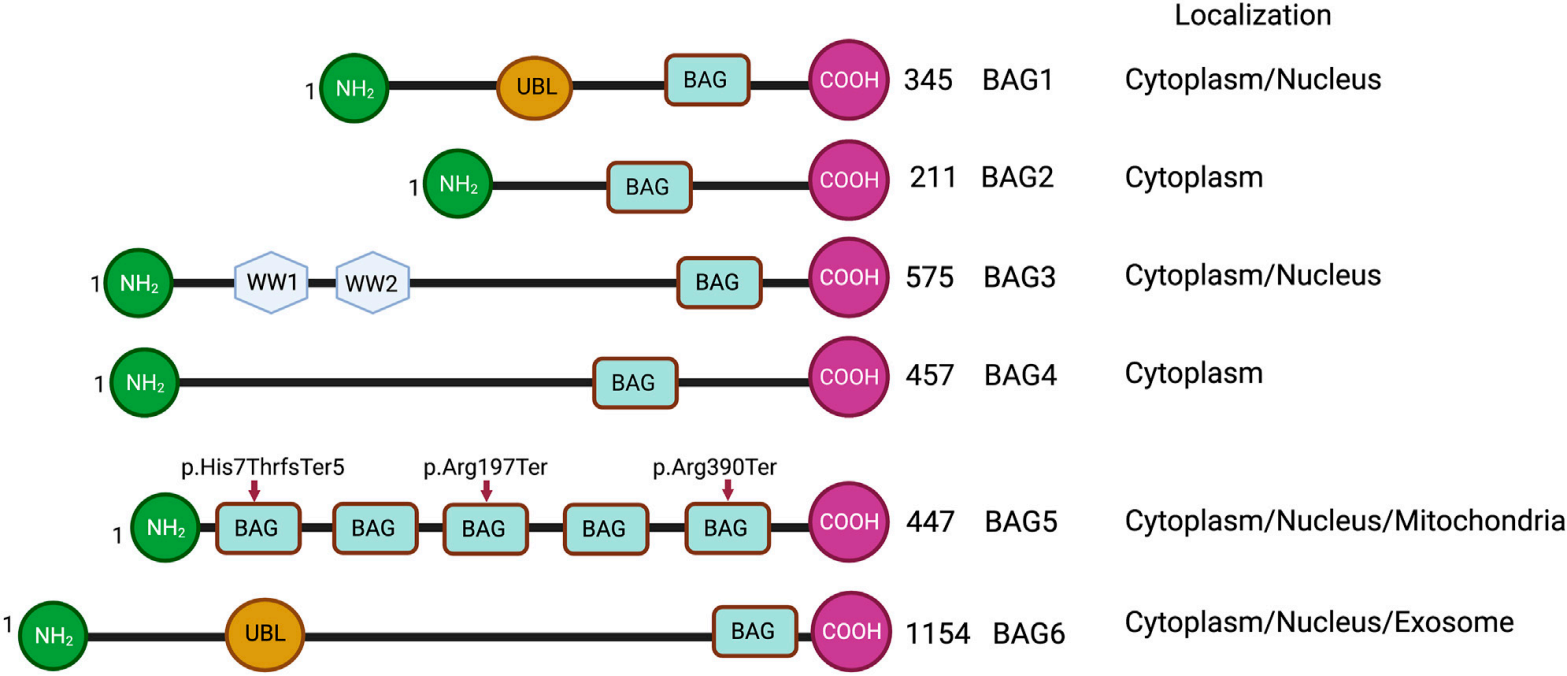
Schematic representations of BAG family proteins, showing conserved domains and cellular localization. NH2 represents the N-terminal and COOH represents the C-terminal of protein. UBL represents a ubiquitin-like domain. BAG represents the BAG domain. WW represents WW domain. Arrowheads show the truncating variants of the BAG5 gene.

**FIGURE 2:**
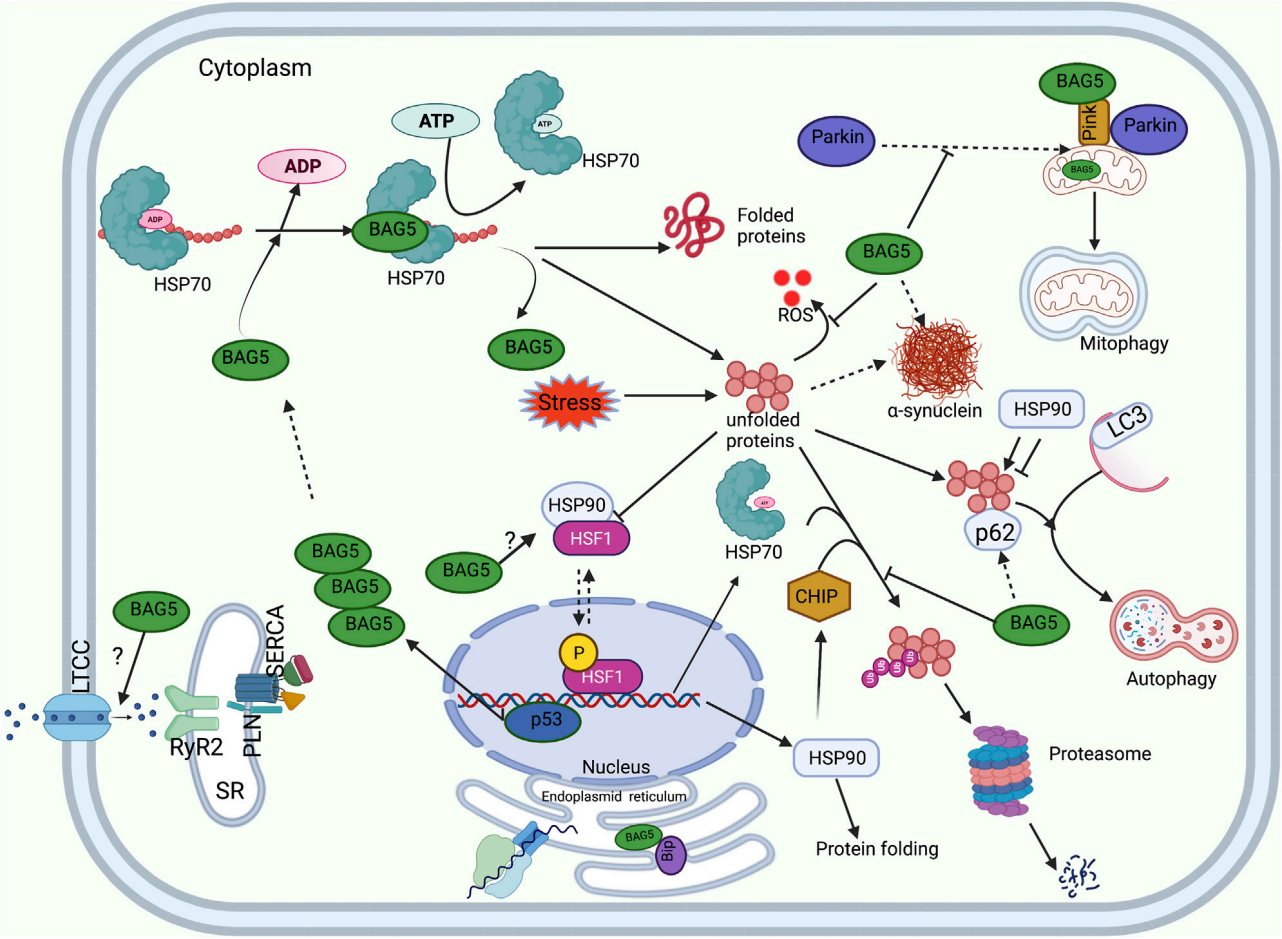
Functions of BAG5 in cellular protein quality control. A diagram shows the involvement of BAG5 in the major cellular functions: regulation of HSP70 mediated protein folding, degradation of protein by autophagy and UPS system, regulation of PINK and Parkin mediated cellular mitophagy, regulation of cellular ER stress, generation of heat shock response, inhibition of ROS production, and handaling of Ca2+ ions. Sarcoplasmic reticulum (SR), L-type calcium channel (LTCC), Ryanodine receptor (RyR), Sarcoplasmic Ca2+ -ATPase (SERCA), Phospholamban (PLN).

Molecular chaperones are the class of proteins that create equilibrium between protein synthesis and degradation in the cell (Arakawa et al., 2010; Friesen et al., 2020). The molecular chaperones and co-chaperones participate in enhancing protein refolding and protecting cells against the buildup of misfolded proteins (Arakawa et al., 2010; Friesen et al., 2020). In the cardiomyocytes, molecular chaperones encompass the major heat shock proteins (HSPs), such as HSP70, HSP90 and co-chaperones, including carboxy terminus of HSP70-interacting protein (CHIP), and BAG family proteins (BAG 1-6) (Figure 1) (Hartl et al., 2011; Balchin et al., 2020). The HSP70 family chaperones are monomeric proteins that are expressed in the heart and play a pivotal role in protecting the cardiomyocytes under stressful conditions (Willis and Patterson, 2010; Ranek et al., 2018). The stress-inducible forms include HSP70-1a, HSP70-1b, HSP70-6, and constitutive forms include HSP70-2, HSP70-5, HSc70, and HSP70-9 (Trcka et al., 2019). Among the inducible forms, HSP70-1a, HSP70-1b is exclusively found in the cytosol, nucleus, and lysosomes, whereas HSP70-6 is usually localized in the cytosol and the nucleus (Trcka et al., 2019). These proteins possess two domains, i.e. ATPase domain and substrate-binding domain (SBD), responsible for regulating their activity. Oxidative stress can induce the expression of HSP70, whereas its protein homolog HSC70 (heat shock cognate) is constitutively expressed in the myocardium (Ranek et al., 2018; Trcka et al., 2019). HSP70 proteins can fold the misfolded proteins to a native protein conformation *via* a ATP-dependent cyclic process and protect cells from proteotoxic stress (Figure 2). However, under physiological conditions, HSP70s facilitate the folding of newly synthesized polypeptides into a typical protein and help in the transport of proteins to the intracellular environment (Figure 2) (Ranek et al., 2018).

HSP 90 proteins are molecular chaperones that stabilize the proteins against heat stress and facilitate their degradation, and are more selective compared to other chaperones in identifying misfolded proteins (Zhao and Houry, 2005; Morán Luengo et al., 2019). These proteins comprise three domains i.e. ATP binding domain, protein-binding domain, dimerizing domain, and they interact with misfolded protein regions of the substrate protein to provide stability to the substrate and reduce the protein aggregation (Figure 2). The members of the HSP90 family include HSP90N, glucose-related protein (GRP) 94 (GRP94), and TNF receptor-associated protein 1 (TRAP1) (Morán Luengo et al., 2019). These chaperones are abundantly expressed in the cytosol and exist in two isoforms i.e. HSP90α (inducible) and HSP 90β (constitutive) (Chen et al., 2005; Morán Luengo et al., 2019). In addition, it has been reported that ischemic conditions cause reactive oxygen species (ROS) build-up, induction of HSP90α, followed by augmentation of HSF1 expression (Ranek et al., 2018).

## Protein Homeostasis in the ER

Apparently, ER-resident chaperones play a significant role in the cellular protein folding and degradation through ERAD pathways. HSP70 member protein, GRP78 also known as BiP, plays a diverse role in maintaining ER-mediated proteostasis. Like Hsp70, GRP78 has SBD and during ER stress, its expression is significantly upregulated to assist the protein folding. Additionally, BiP plays a significant role in maintaining the translocation of proteins in the ER by retrograde transport of misfolded proteins for degradation through the ERAD system (Pobre et al., 2019). Intriguingly, ER is equipped with enzymes and chaperones that assist in the folding and assembly of proteins. The folding of proteins in a proper way requires the formation of an intramolecular or intermolecular disulfide bond in the native proteins to achieve the proper structure. ER-resident protein disulfide isomerase (PDI) plays a critical role in crosslinking of native proteins via disulfide bond formation. PDI expression is found to be upregulated during ER stress, and overexpression of PDI protects cells from ER stress induced cell death (Feige and Hendershot, 2011; Shin et al., 2021). Another member of heat shock protein HSP90, known as a GRP94 found to be in the ER. The chaperonic activity of GRP94 is restricted to selective proteins. It mainly assists the folding of membrane-associated proteins or secretory proteins (Feige and Hendershot, 2011; Shin et al., 2021). Most of the proteins that form in the ER undergo post-translational modification by forming an N-glycosidic bond with the glycans N- (carbohydrate-based polymers). This PTM help in protein folding, translocation, solubilization, and degradation. Apart from this, ER proteins calnexin and calreticulin also act as chaperones for the glycoprotein and helps in protein folding and terminal release of proteins (Ihara et al., 2020; Kozlov and Gehring, 2020).

## HSF1 Mediated Regulation of Cellular Proteostasis

Interestingly, the expression of chaperones is regulated by transcription factors HSF1 and HSF2 (Willis and Patterson, 2010). HSF1 transcription factor is present in the cytosol as a monomer and binds to HSP90 to impede its chaperone activity (Willis and Patterson, 2010). However, under stressful conditions, denatured proteins bind HSP90 and release HSF1, which then translocate to the nucleus to bind to the heat shock response elements of the stress-induced genes (promoter region) (Willis and Patterson, 2010). In the nucleus, augmented levels of HSF1 up-regulate the expression of HSP70 chaperone. After that, HSP70 binds HSF-1 to reduce its activity via the feedback mechanism. Apart from this, HSF-1 dependent stress response causes up-regulation of ubiquitin such that it enhances the ability of the cell to degrade proteins under stressful conditions (Willis and Patterson, 2010). Interestingly, a recent study indicated that HSF-1 regulates the expression of BAG family proteins as well (Prince et al., 2020). In fact, it was shown that the expression of HSF1 protein and its nuclear localization shows a positive correlation with BAG3 expression in the human cardiac tissue (Martin et al., 2021a). Previous studies have indicated that the expression of HSP90 is induced during cardiac arrhythmia, cardiac remodeling associated with cardiac hypertrophy, and heart failure (Ranek et al., 2018). This indicates that HSP90 proteins play a crucial role in the maturation of proteins and the progression of cardiac disorders (Figure 2). Interestingly, it was demonstrated that BAG5 protein strongly interacts with HSF1. However, the functional significance of BAG5 and HSF1 interaction needs to be established (Taipale et al., 2014).

## BAG Protein Family and Its Molecular Interaction with HSP70 and Other Cellular Proteins

The BAG family is a set of multifunctional proteins that play a pivotal role in maintaining cellular homeostasis (Arakawa et al., 2010; De Snoo et al., 2019). The human BAG family comprises of six members viz. BAG1, BAG2, BAG3, BAG4, BAG5 and BAG6 (Arakawa et al., 2010; Friesen et al., 2020) (Figure 1) and found to be localized in nucleus, cytoplasm, mitochondria as well as in exosome (Figure 1). The members of this family contain a unique BAG domain which facilitates their interaction with the ATPase domain of molecular chaperone heat shock protein (HSP70) (Arakawa et al., 2010). Interestingly, BAG3 protein has some additional domains like WW domains in addition to the BAG domain, which helps in interacting with other proteins (Merabova et al., 2015; Sherman and Gabai, 2022). BAG family proteins play a significant role in regulating several physiological functions, like autophagy, UPS mediated protein degradation, and apoptosis (Kögel et al., 2020). Among the family members, BAG5 is considered exceptional as it comprises five domains in tandem. It has been reported that the fifth domain (BD5) is quintessential for exhibiting the interaction between HSP70 and BAG5 (Arakawa et al., 2010). Furthermore, it is manifested that the binding of BD5 induces certain conformational changes in the nucleotide-binding domain (NBD) of HSP70 such that it loses its affinity for binding ADP. In an *in-vitro* assay, it was found that BD5 or full-length BAG5 acts as a nucleotide exchange factor of HSP70 and accelerates the protein refolding activity of HSP70 (Figure 2) (De Snoo et al., 2019).

Interestingly, among all the members of the family, BAG5 has drawn considerable attention as it is expressed in the cardiomyocytes (Gupta et al., 2016) and plays a pivotal role in monitoring cell homeostasis under stressful conditions (Gupta et al., 2016; Duggan et al., 2021). In fact, studies have indicated that BAG5 protein overexpression can limit oxidative stress and mitochondrial dysfunction in the cells (Wang et al., 2014; Gupta et al., 2016).

## BAG Family and Its Association with Cardiac Disease

BAG-family proteins participate in various cellular processes, including cell survival, migration, and proliferation (Rosati et al., 2011). Interestingly, over-expression of BAG-family proteins has been found in various cancers and is known to promote cell survival and induce cell proliferation. However, these proteins also possess anti-apoptotic activity, which depends on their interactions with either Hsc70/Hsp70 protein or binding to Bcl-2 protein (Doong et al., 2002; Rosati et al., 2011). Anticancer drug JG-98 is found to be effective in reducing the cancer cells growth by inhibition of BAG3-HSP70 interaction (Martin et al., 2022). However, this drug induces cardiac toxicity through reduction of BAG3 proteins half-life and reduction of cellular autophagy (Martin et al., 2022).

BAG family proteins are expressed in various tissues, including the heart (Gupta et al., 2016; Qin et al., 2017), lungs, brown adipose tissue (Qin et al., 2016), and are associated with various cellular components, including endoplasmic reticulum (Saxena et al., 2012), mitochondria (De Snoo et al., 2019; Li et al., 2010), microtubules (de Paula et al., 2016; He et al., 2020). In addition, BAG family proteins play a pivotal role in maintaining the structural and functional integrity of the heart (Knezevic et al., 2015). In fact, mutations in these proteins may result in structural as well as functional defects in the heart such that it can make an individual more vulnerable to CVDs, including heart failure (Knezevic et al., 2015; Diofano et al., 2020). A recent study shows that cardiac patients having truncation mutations (p.Arg197Ter and p. His7ThrfsTer5) of the BAG5 gene develop tachycardia-induced cardiomyopathy (Figure 1). Furthermore, this study shows that mouse models with the BAG5 gene mutation (Arg197Ter) have ventricular dilatation and dysregulation of calcium handling with arrhythmogenicity, suggesting that BAG5 is important for cardiac function (Hakui et al., 2022).

Apparently, studies have indicated that the BAG family proteins play a crucial role in mediating the adaptive cell survival response against sustained ischemia and thereafter reperfusion via induction of autophagy (Gurusamy et al., 2009). It has been reported that BAG-1 protein works in consensus with LC3-II (autophagosomal protein) and HSC70 protein to induce autophagy (Gurusamy et al., 2009). Also, it has been shown that BAG-3 protein participates in inducing autophagy in association with HSPB8 protein. A recent study shows that reduction of BAG3 mediated protein turnover may cause cardiomyocytes’ contractile dysfunction during heart failure (Martin et al., 2021b). Apparently, this indicates that BAG family proteins promote cell survival under stressful conditions via induction of autophagy (Kögel et al., 2020).

## Role of BAG5 in Modulating Mitophagy

Cardiac muscle cells are constantly beating to maintain heart function, and these cells have high energy demand and are therefore, rich in mitochondria content compared to other types of cells. Mitochondria provide most of the cellular energy through oxidative phosphorylation, and maintaining a healthy mitochondrial pull is important for cardiac health (Murphy and Hartley, 2018). Under mild stress conditions, mitochondrial components can deteriorate, and mitochondria can revive by the process of mitochondrial fusion or fission event and maintain the cellular energy demands (Wai et al., 2015; Murphy and Hartley, 2018). However, under severe stress conditions, mitochondria cannot revive and need to be removed from the cell to maintain a healthy cellular condition. Mitochondrial protein quality control (MQC) is the most vital process for maintaining mitochondrial homeostasis, removing dysfunctional or superfluous mitochondria such that the cells are shielded against oxidative stress (Picca et al., 2018; Bakula and Scheibye-Knudsen, 2020). Damaged mitochondria can be removed from the cell by a vesicular process, where mitochondria with the help of some chaperones are engulfed into a double membrane structure and then fused with the lysosome for degradation and recycling of degraded materials. Several studies suggest that dysfunctional MQC accumulates damaged mitochondria and generates ROS production, leading to the progression of several diseases, including CVD, Alzheimer’s disease, and Parkinson’s disease (Kornfeld et al., 2015; Kolodkin et al., 2020; Wenzhang Wang et al., 2020). PTEN-induced kinase 1 (PINK1) and Parkin play an important role in maintaining healthy mitochondrial pull and regulation of MQC (Gong et al., 2015).

Accumulating evidence from different studies suggests that PINK1 plays a dual role in the regulation of MQC (Ge et al., 2020). PINK1 is a mitochondrial serine/threonine-protein kinase that undergoes post-translational cleavage in the inner mitochondrial matrix and generates a c-terminal PINK1 (c-PINK1). Previous studies suggest that c-PINK1 in association with cellular chaperone can stay in the cytosol or be degraded through the UPS system. It was demonstrated that c-PINK is important for the biogenesis of mitochondria as well as to protect the healthy mitochondrial pull in the cytosol. c-PINK inhibits the Parkin protein translocation to the mitochondria as well as degradation of Parkin by the UPS system. Additionally, c-PINK can protect the mitochondria from mitophagy by promoting the phosphorylation of LC3 and upregulation of the mTORC2 pathway (Nan Wang et al., 2020). However, during severe stress and irreversible mitochondrial damage, PINK1 promotes mitophagy to remove damaged mitochondria from the cell. During stress, PINK1 is not processed by the mitochondria, and unprocessed PINK1 accumulates on the outer mitochondrial membrane (OMM) of the damaged mitochondria. Membrane-associated PINK1 can undergo autophosphorylation as well as phosphorylate the ubiquitin molecule tagged with the OMM proteins. These changes promote the translocation of the Parkin protein to the damaged mitochondria and ubiquitinate the mitochondrial surface proteins. Ubiquitinated proteins can be recognized by the mitophagy adaptor proteins like P62, LC3 and undergo the mitophagy process (Nan Wang et al., 2020).

Earlier studies have reported that the physical interaction between PINK1 and BAG5 plays a pivotal role in modulating PINK1 protein degradation (Wang et al., 2014). It was shown that BAG5 overexpression led to a marked increase in the level of PINK1 protein accompanied by a reduction in PINK1 degradation. Also, BAG5 knockdown significantly reduced PINK1 levels, which was associated with increased PINK1 ubiquitination. This indicated that BAG5 monitors the PINK1 level and abrogates its degradation via stabilizing PINK1 protein and inhibiting Parkin E3 ligase activity (Wang et al., 2014). Apart from this, it was also demonstrated that the decline in the PINK1 level or suppression of PINK1 expression could lead to a significant increase in the BAG5 level and increased localization of BAG5 protein in the mitochondria. Probably increased expression of BAG5 in PINK1-suppressed cells is a compensatory feedback regulatory mechanism of cells (Wang et al., 2014). Besides, the authors reported that administration of MPP^+^ (neurotoxin) induced mitochondrial dysfunction (loss of mitochondrial membrane potential, cytochrome c release, augmentation in ROS level) was overcome by increasing the expression of BAG5 protein and subsequent upregulation of endogenous PINK1 protein (Wang et al., 2014). Furthermore, another group of researchers reported that MPP^+^ (1-methyl-4-phenylpyridinium, neurotoxin)-administration induced apoptosis in PC12 cells (cells derived from rat adrenal medulla). However, BAG5 overexpression protected the PC12 cells against MPP^+^-induced apoptosis by significantly upregulating various proteins, including Bcl-2 (anti-apoptotic protein) and Bcl-xl, decreasing cleaved caspase-3 level, reducing cytochrome c release, and inactivating downstream signaling cascade of apoptosis (Ma et al., 2012).

Another study reported that under mild stress, Parkin-dependent mitophagy is minimized as BAG5 impedes recruitment of Parkin into the depolarized mitochondria in the human osteosarcoma U2OS cell line. Meanwhile, simulation of chronic stress *via* dissipation of mitochondrial membrane potential with carbonyl cyanide m-chlorophenyl hydrazine (CCCP), BAG5 promoted apoptosis by enhancing Parkin-dependent Mcl-1 (pro-survival factor) polyubiquitination and degradation in SH-SY5Y neuroblastic cells. However, knockdown of BAG5 partially reduced Parkin-dependent Mcl-1 degradation in CCCP treated cells. Also, BAG5 overexpression-dependent enhanced cell death was overcome by knocking out Parkin using CRISPR/Cas. This indicates that Parkin interacts with BAG5 and other chaperone proteins during stressful conditions, which can promote Parkin’s switching from pro-survival to the pro-apoptotic pathway (De Snoo et al., 2019).

A recent study with rat cardiomyocytes by Tan et al. showed that Hexokinase-II (HK-II) could play an important role in MQC. HK-II can sense metabolic disturbance of cells and modulates its intracellular localization to modulate mitophagy and cell survival. It has been reported that administration of mitochondrial HK-II (mitoHK-II) dissociating peptide reduces mitochondrial HK-II levels but increases Parkin translocation to the mitochondria and the ubiquitination of mitochondrial proteins in the cardiomyocytes. Interestingly, BAG5 owes the ability to localize to mitochondria and form a complex with HK-II. The exposure of the cardiomyocytes to an ischemic condition induced modest dissociation of mitoHK-II. In fact, the enhancement of this process protected the cardiomyocytes against ischemic damage via inducing mitophagy. The authors reported that BAG5 overexpression inhibits HK-II translocation from mitochondria and protects the mitochondria from Parkin-mediated mitophagy. This study suggests that BAG5 is an important co-chaperone of the HK-II and helps in maintaining an optimum level of mitophagy in basal conditions (Tan et al., 2019).

## Role of BAG5 in Regulating Endoplasmic Reticulum Stress and Cell Viability

Our previous study indicated that during endoplasmic reticulum (ER) stress, unfolded proteins accumulate, which activates unfolded protein response (UPR). To counter the UPR during ER stress, the cells might upregulate the expression of ER stress-related chaperone and co-chaperone proteins to facilitate the protein folding capacity of ER and subsequently reduce ER stress (Ren et al., 2021). We reported that tunicamycin (antibiotic to induce endoplasmic reticulum stress) administration in the cardiomyocytes was associated with an enhancement in the expression of ER-associated proteins, i.e., GRP78, and pro-apoptotic C/EBP homologous protein (CHOP). Additionally, it was found that during tunicamycin-mediated ER stress, BAG5 expression increased in a time-dependent manner in the cardiomyocytes, and the interaction between BAG5 and GRP78 was considerably increased. It is manifested that GRP78 elicits protective effects against ER stress either by acting as a molecular chaperone or reducing the load of UPR. It was seen that during ER stress, the interaction of BAG5 with GRP78 was significantly increased such that it tended to stabilize GRP78. Intriguingly, it was found that BAG5 protein overexpression considerably reduced cell death and improved the viability of cardiomyocytes via reducing the expression of CHOP and caspase-3 (Gupta et al., 2016). This indicates that BAG5 plays a protective role against stress via improving the survival of cardiomyocytes (Gupta et al., 2016). However, knockdown of BAG5 remarkably increased cell death and reduced cell viability in the cardiomyocytes. This indicates that BAG5 exhibits protective effects against endoplasmic stress during stressful conditions by modulating the expression of stress-induced CHOP and GRP78 proteins (Figure 2) (Gupta et al., 2016).

Similarly, another recent study reported that BAG5 acts as an ER stress regulator and its expression contributes to maintaining endothelial cell viability and protects cells from endothelial dysfunction (Zhu et al., 2021). This study found that catecholamine-dependent endothelial dysfunction immensely causes an increase in oxidative stress via augmenting ROS production and decreasing the levels of antioxidant factors viz. superoxide dismutase, glutathione, and glutathione peroxidase. The study unraveled that BAG5 overexpression could overcome catecholamine-dependent endothelial dysfunction and improve cell survival *via* decreasing oxidative stress and improving endothelial cell survival. Mechanistically, it was found that catecholamine treatment significantly increases protein kinase R-like endoplasmic reticulum kinase (PERK) and activates transcription factor 6 (ATF6) levels in the endothelial cells. Apparently, PERK and ATF6 are endoplasmic stress transducers and induce either apoptosis or autophagy. It was found that BAG5 overexpression considerably reduced the level of PERK and ATF6 in the endothelial cells indicating that the endoplasmic stress was negatively correlated to BAG5 expression. Also, it was found that catecholamine treatment inhibits ERK activity in the cells, accompanied by a reduction in BAG5 transcription. Intriguingly, MAPK-ERK activation using a pharmacological agonist reversed the decline in BAG expression in the dysfunctional cells. This indicated that catecholamine-dependent BAG5 downregulation was possible because of MAPK-ERK inactivation and reactivation of MAPK-ERK abrogated the endoplasmic reticulum stress, oxidative stress, and improved the cell survival via modulating MAPK/ERK signaling and BAG5 transcription (Zhu et al., 2021). The role of BAG5 as a stress regulator is further substantiated by a very recent study which revealed that exposure of primary neuronal cultures to H_2_O_2_ for 6 h induced oxidative stress. Also, this was accompanied by a reduction in the expression of BAG5 protein (Duggan et al., 2021). This indicates that stressful conditions downregulate BAG5 expression and induce cell death.

## Role of microRNAs in Regulating BAG5 Expression

A previous study showed that microRNA miR-155 is involved in various pathological processes including CVDs (Gangwar et al., 2018). Xi et al. reported that exposure of H9C2 cells to hypoxia and thereafter, reperfusion led to a significant increase in cell death, accompanied by an increase in the expression of miR-155. Interestingly, the increase in the expression of miR-155 was accompanied with downregulation in the expression of BAG5 protein. Further, the authors found that transfection of miR-155 mimic into the H9C2 cells also reduced BAG5 protein expression and cell viability. This indicated that miR-155 negatively regulated BAG5 protein expression and cell viability. However, the authors also found that inhibition of miR-155 reduced myocardial apoptosis, improved cell injury, and this protective effect was further promoted by BAG5 protein overexpression. Also, the inhibition of miR-155 was followed by reduced JNK/MAPK signaling. This indicated that miR-155 modulated myocardial hypoxia-reperfusion injury via regulating the JNK/MAPK signaling and the downregulation of BAG5 expression (Xi et al., 2020).

This is further corroborated by a recent study where miR-155 (siRNA) transfection into UE6E7T-2 cells (human mesenchymal stem cell) disrupted mitophagy and interfered with mitochondrial PQC in the cells. The authors found that miR-155 directly reduced BAG5 expression and considerably augmented the ubiquitination and degradation of PINK1 protein for clearing the damaged mitochondria. This indicated that BAG5 expression regulates ubiquitination status and a decline in its expression results in exaggerated ubiquitination of the mitochondrial proteins (Tsujimoto et al., 2020). Thus, microRNAs modulate BAG5 expression, and its interaction with PINK1 plays a critical role in maintaining mitochondrial homeostasis under stressful conditions. Additionally, it was demonstrated that miR-4454 and miR-127 suppress the cancer cells through regulation of BAG5 expression and over expression of BAG5 can neutralize the suppressive effect of miR-127 and miR-4454 (Bi et al., 2016; Dasari et al., 2020).

## Role of BAG5 in the Regulation of the mTOR Pathway

A recent study published by Wang et al. showed that expression of BAG5 significantly decreased in the cisplatin-resistant ovarian cancer tissue, and down regulation of BAG5 made the cancer cells chemoresistant (Wang et al., 2021). Further, the authors found that knockdown of the BAG5 causes metabolic reprogramming and maintenance of cancer stem cells like feathers of the ovarian cancer cells. Mechanistically, it was demonstrated that Bcl6 protein binds to the promoter of the BAG5 gene and causes transcriptional suppression of BAG5 gene expression. Additionally, it was demonstrated that suppression of BAG5 may cause the upregulation of the rictor-mTORC2 pathway which modulates cellular metabolism and cancer stem cell-like feathers of cisplatin-resistant ovarian cancer cells. Furthermore, the authors concluded that BAG5 could be useful as a novel target to control cancer cell growth. However, the role of BAG5 in the regulation of autophagy through mTOR pathway needs to be investigated.

## Role of BAG5 in Parkinson’s Disease

Astonishingly, some studies have reported that BAG5 interaction with other chaperones might interfere with their E3 ubiquitin ligase activity such that it might cause accumulation of toxic oligomers of α-synuclein (α-syn) (Chen et al., 2020). Parkinson’s disease is a neurodegenerative disorder characterized by abnormal protein homeostasis causing accumulation of the protein α-syn in the form of Lewy bodies (Orme et al., 2018; Goldman et al., 2020). Previous studies have indicated that the carboxyl terminus of HSP70-interacting protein (CHIP, a co-chaperone) exhibits E3 ubiquitin ligase activity such that it may hamper the accumulation of toxic α-syn oligomers (Kalia et al., 2011). The authors unveiled that in the brain, CHIP, α-syn, and BAG5 interact *via* HSP70 protein.

CHIP comprises of tetratricopeptide repeat (amino-terminal), which facilitates its interaction with HSP70/90, whereas the U-box domain (carboxy-terminal) promotes the interaction with BAG5. The interaction of BAG5 with CHIP protein (Carboxy terminus of HSP-70 Interacting protein) impedes its E3 ubiquitin ligase activity in human H4 neuroglioma cells. This results in reduced α-synuclein ubiquitinylation and increases the propensity for α-synuclein to oligomerize and form intracellular protein aggregates (Lewy bodies), which are toxic to the cells. This is further supported by the fact that knockdown of CHIP promoted α-synuclein oligomerization, indicating that CHIP-dependent ubiquitinylation reduces the formation of α-synuclein oligomers (Kalia et al., 2011). Additionally, another study demonstrated that oxidative stress inducers like H2O2 and etoposide could induce the expression of transcription factor p53. Oxidative stress-induced P53 binds to the promoter region of the BAG5 gene and causes upregulation of BAG5 protein expression. Also, it was shown that colocalization of BAG5 and α-synuclein significantly increases in the cells which might result in α-synuclein aggregation. This suggests that BAG5 protein may play an important role in the progression of Parkinson’s disease (Chen et al., 2020).

Apart from the Parkin gene, DJ-1 is another pathological gene involved in the onset of Parkinson’s disease. DJ-1 is a multifunctional protein with an antioxidant property and acts as a molecular chaperone to maintain cellular homeostasis. Under normal physiological conditions, DJ-1 localizes in the mitochondria and monitors its activity *via* binding to subunits of mitochondrial complex I. However, during oxidative stress, DJ-1 dimerizes and with the help of chaperones, translocates to the mitochondria to prevent oxidative stress-induced cell death. In fact, DJ-1 interacts with proteins including Parkin, PINK1, and HSP70 to protect the cells against oxidative stress-induced disorders. Interestingly, it has been reported that the interaction of BAG5 and DJ-1 protein may enhance apoptotic cell death in neurodegenerative disorders, including Parkinson’s disease. Qin et al. showed that exposure of HEK293 cells to rotenone (inhibitor of complex I of the mitochondrial respiratory chain) co-expressing BAG5 and DJ-1 significantly increased the ROS production in comparison to the cells expressing DJ-1 alone. This indicated that the protective effects of DJ-1 against rotenone-induced cell apoptosis are abolished in the presence of BAG5 protein. Further, the authors reported that overexpression of BAG5 in HEK293 cells decreases DJ-1 levels indicating that BAG5 inhibits its dimerization and mitochondrial translocation. However, this effect could be reversed by overexpressing HSP-70 protein. This suggests that HSP-70 and BAG5 protein act in concert to regulate the stability of DJ-1 protein and subsequently regulate cell viability (Qin et al., 2017).

## Role of BAG5 in Alzheimer’s Disease

Various studies have reported that the expression of BAG5 protein in the brain monitors the development of neurodegenerative diseases (Kalia et al., 2011; Guo et al., 2015). Using human neuroblastoma cells (SH-SY5Y), Guo and co-workers reported that BAG5 expression is modulated in Alzheimer’s disease. Furthermore, the authors revealed that BAG5 expression at both transcriptional and translational levels is upregulated in the transgenic mice suffering from Alzheimer’s disease. In fact, *in-vitro* experiments revealed that administration of Aβ1-42 (10 µM) increased the expression of BAG5 in a dose-dependent manner. However, inhibition of BAG5 expression using siRNA exaggerated ROS generation, augmented malondialdehyde levels, and promoted Aβ1-42-mediated neurotoxicity. This was also accompanied by caspase-3 cleavage and elevation in the number of apoptotic cells (Guo et al., 2015). However, further research is needed to understand the role of BAG5 in neurodegenerative diseases.

## Conclusion

It is now believed that aging is the most significant risk factor associated with CVDs, neurological disorders, inflammation, metabolic abnormality, and drug toxicity which causes premature death worldwide. The past 2 decades of research have generated several novel findings and determined the risk factors associated with aging. It was noticed that dysregulation of cellular proteostasis could cause formation of cellular protein aggregates, which can lead to the development of CVDs and neurological disorders. Autophagy and UPS are two important cellular PQC systems, which remove the unfolded or aggregated proteins from the cell and maintain a clean cellular environment. Mitochondria plays a vital role in the supply of high energy demands in the muscles and brain. Environmental stress can affect mitochondrial function and lead to ROS production through stressed mitochondria. Although, mitochondrial biology is known, we are still not clear about the role of chaperones and co-chaperones in maintaining healthy mitochondria in the cells. BAG5 protein plays a critical role in maintaining cellular PQC under normal and stressful conditions. Additionally, BAG5 can regulate the turnover of mitochondria during normal and stressful conditions. Earlier studies suggest that BAG5 can promote cell death, but recent studies from own laboratory as well as from other laboratories show that BAG5 can protect cells from oxidative stress and modulate autophagy in the cell to improve cell survival. Also, micro RNAs can regulate the expression of BAG5 protein to limit cell apoptosis and subsequently death. Since BAG5 protein expresses in the heart and brain, it has become a novel target that can be exploited for the development of treatment strategies for curing cardiovascular and cerebrovascular disorders.
